# Carnivoran Remains from the Malapa Hominin Site, South Africa

**DOI:** 10.1371/journal.pone.0026940

**Published:** 2011-11-03

**Authors:** Brian F. Kuhn, Lars Werdelin, Adam Hartstone-Rose, Rodrigo S. Lacruz, Lee R. Berger

**Affiliations:** 1 Institute for Human Evolution, University of the Witwatersrand, Johannesburg, South Africa; 2 School of GeoSciences, University of the Witwatersrand, Johannesburg, South Africa; 3 Department of Palaeozoology, Swedish Museum of Natural History, Stockholm, Sweden; 4 Pennsylvania State University Altoona, Altoona, Pennsylvania, United States of America; 5 Center for Craniofacial Molecular Biology, University of Southern California, Los Angeles, California, United States of America; University College London, United Kingdom

## Abstract

Recent discoveries at the new hominin-bearing deposits of Malapa, South Africa, have yielded a rich faunal assemblage associated with the newly described hominin taxon *Australopithecus sediba*. Dating of this deposit using U-Pb and palaeomagnetic methods has provided an age of 1.977 Ma, being one of the most accurately dated, time constrained deposits in the Plio-Pleistocene of southern Africa. To date, 81 carnivoran specimens have been identified at this site including members of the families Canidae, Viverridae, Herpestidae, Hyaenidae and Felidae. Of note is the presence of the extinct taxon *Dinofelis* cf. *D. barlowi* that may represent the last appearance date for this species. Extant large carnivores are represented by specimens of leopard (*Panthera pardus)* and brown hyaena (*Parahyaena brunnea*). Smaller carnivores are also represented, and include the genera *Atilax* and *Genetta,* as well as *Vulpes* cf. *V. chama.* Malapa may also represent the first appearance date for *Felis nigripes* (Black-footed cat). The geochronological age of Malapa and the associated hominin taxa and carnivoran remains provide a window of research into mammalian evolution during a relatively unknown period in South Africa and elsewhere. In particular, the fauna represented at Malapa has the potential to elucidate aspects of the evolution of *Dinofelis* and may help resolve competing hypotheses about faunal exchange between East and Southern Africa during the late Pliocene or early Pleistocene.

## Introduction

The South African palaeontological record of the Plio-Pleistocene is rich and diverse. Most deposits from this time period are constrained within a relatively small geographical area in the Sterkfontein Valley (Cradle of Humankind). These cave sites range in age from ca. 2.5 Ma to the Holocene. The analyses of faunal remains from these sites have consistently revealed a change in ecology from the older Sterkfontein site (ca. 2.5 Ma), hosting the largest record of the hominin *Australopithecus africanus* and characterized by relatively heavy tree cover [1], to younger deposits (Kromdraai, Swartkrans, Drimolen, Coopers) which commonly include the hominin taxa *Paranthropus* and *Homo* and are characterized by more open habitats [1], [2]. These latter sites provide evidence of faunal evolution in the region generally within the period between 1.8 Ma and 1.5 Ma. Historically, the period from approximately 2.0 Ma to 1.8 Ma has remained largely unsampled in southern Africa. This is a critically important time period as it encompasses the ecological shift from faunas typified by assemblages such as that of Sterkfontein, associated with *Au. africanus*, to the faunas and ecology of the younger *Paranthropus/Homo*-bearing deposits. The recent discovery of *Australopithecus sediba*, the youngest known *Australopithecus* species dating to 1.977 Ma [3], and associated faunal remains provide a unique window to interpreting faunal changes and ecology in southern Africa during this critical time period. This contribution focuses on the analysis of the carnivoran remains recovered from this site to date, paying particular attention to the *Dinofelis* specimens.

The site of Malapa was discovered in August of 2008 during a survey of cave sites in the region. The geological setting of Malapa is unique when compared to the majority of sites in the region; it is thought to have been a death trap based on the lack of carnivore-damaged bones and the high number of articulated specimens [4]. At present, Malapa appears as a deroofed cave deposit with an exposed area of approximately two metres by one and half metres by two metres deep. Cosmogenic analyses suggest that the site today is the bottom of what would have been a very deep cave system 1.9 million years ago [4]. Malapa has produced over 130 specimens attributed to the new hominin species *Australopithecus sediba* [5]. In addition to the hominin specimens, the site has produced a diversity of bovid and carnivore taxa that enable us to suggest a biochronological date of between 2.36 and 1.5 Ma [4]. Uranium-lead (U-Pb) dating and palaeomagnetic studies further constrain the likely deposition of the sediments to 1.977 Ma [3].

## Results

To date 81 specimens have been attributed to the order Carnivora (Table 1). Of these, 69 have been identified to at least the family level and are described here. The remainder are listed as Carnivora indet.

**Table 1:** List of carnivore remains identified to date with MNI and NISP.

Order	Family	Genus and Species	MNI	NISP
Carnivora	Felidae	*Dinofelis barlowi*.	1	1
		cf. *Dinofelis* sp.	1	15
		*Panthera pardus*	1	1
		*Panthera* cf. *P. pardus*	1	1
		cf. *Panthera sp.*	2	3
		*Felis nigripes*	1	1
		Felidae indet.	1	13
	Hyaenidae	*Parahyaena brunnea*	1	6
		cf. *Parahyaena brunnea*	2	10
		Hyaenidae indet.	1	8
	Canidae	*Vulpes* cf. *V. chama*	1	3
		Canidae indet. (Large)	1	1
	Herpestidae	*Atilax* cf. A. mesotes*	1	1
		cf. Herpestidae	1	4
	Viverridae	cf. *Genetta* sp	1	1
Carnivora		Indet.	1	12
Total			18	81
*Considered by some to be *Herpestes mesotes*				

Family CANIDAE Fischer, 1817

Genus *Vulpes* Frisch, 1775

Vulpes chama Smith, 1833

*Vulpes* cf. *V. chama*

Material

**Figure 1:**
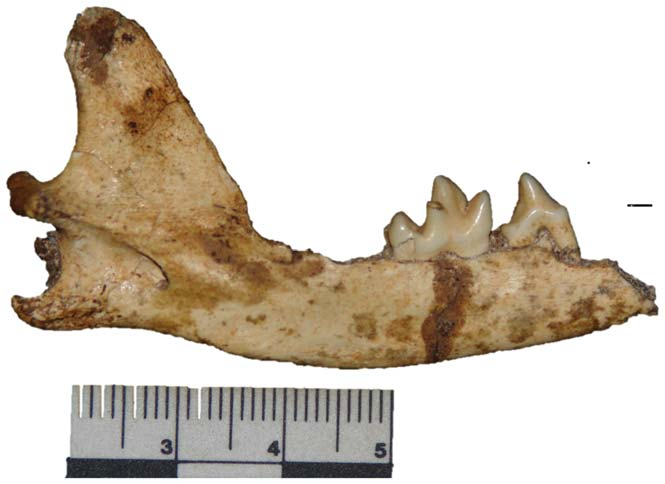
A lateral view of the specimen UW 88–812, a mandible of *Vulpes* cf. *V. chama,* recovered from Malapa. Note the lack of a distal accessory cusp on the P_4._

UW 88–812, partial left mandible with P_4_-M_1_, alveoli for M_2_ and M_3_ (Figure 1); UW 88–813, first rib; UW 88–814, isolated M_2_ L 6.34 mm W 5.27 mm.

Comments

Specimen UW 88–812 is tentatively identified as *Vulpes* cf. *V. chama* based on M_1_ morphology and the presence of M_2_ and M_3_ alveoli. The corpus is low and slender, with the coronoid process rising at about 45° posterior to the M_3_ alveolus. The P_4_ is tall and compressed. It has no mesial accessory cusp. The main cusp is sharply pointed as is normal in *Vulpes*. However, unlike all other specimens of *Vulpes* we have seen, it lacks a distal accessory cusp, there being only a very low crest in its place on the distal face of the main cusp. The M_1_ is typically canid, with a short, low paraconid and taller protoconid. The metaconid is nearly the same height as the paraconid and set slightly posterior to the protoconid.

Compared to other small fossil and extant Canidae our material clearly differs from *Otocyon* in both size and morphology. It further differs from *V. pulcher* and *V. pattisoni* in being smaller. It is most similar to the extant *V. chama*, but features such as the lack of a distal accessory cusp on P_4_ make us hesitant to ascribe it to this species.

The other two specimens listed were found in association with the mandible. UW 88-814 matches *V. chama* in morphology, whereas UW 88–813 is placed here because it represents a small canid and because of the association with the other two specimens. All are likely to belong to a single individual.

Canini indet. (Large species)

Material

UW 88–838, right distal femur (incomplete).

Comments

**Figure 2:**
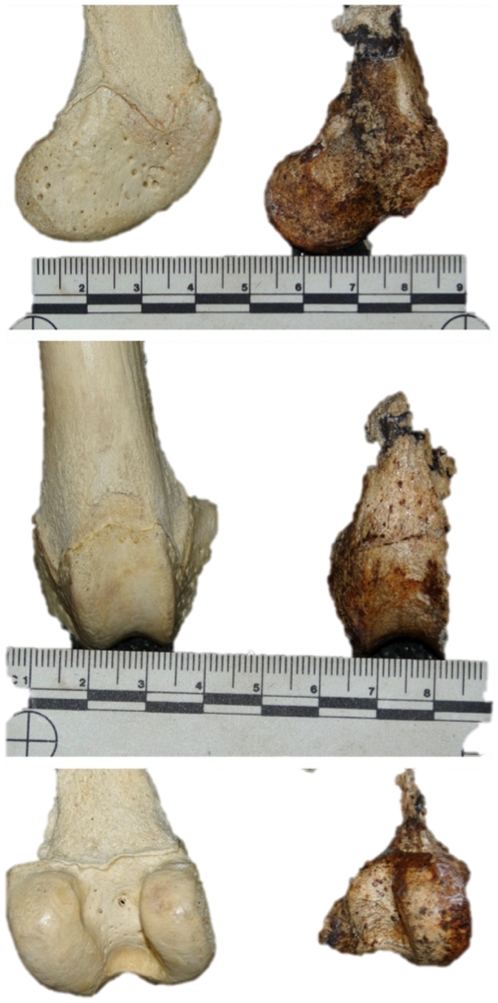
The lateral, cranial and caudal views of specimen UW 88–838, a distal femur of a canini, recovered from Malapa compared to the distal femur of extant *L. pictus*.

While this specimen is definitely from the family Canidae and judging solely by size belongs in the tribe Canini, it would appear not to be the extant wild dog (*Lycaon pictus*) although it is of similar size (Figure 2). Interestingly, it appears similar in size to the Gladysvale specimen (GV 466) that has been attributed to *Lycaon sekowei* [6], but since there is no overlap in skeletal representation between the specimens, no specific attribution can be made. More material is required before we can make an assignation beyond the family level.

Family VIVERRIDAE, Gray 1821

Subfamily VIVERRINAE, Gray 1821

Genus *Genetta* Cuvier, 1817

cf. Genetta sp.

Material

UW 88–834, right mandible fragment with P_4_, M_1_, and tooth roots.

Comments

**Figure 3:**
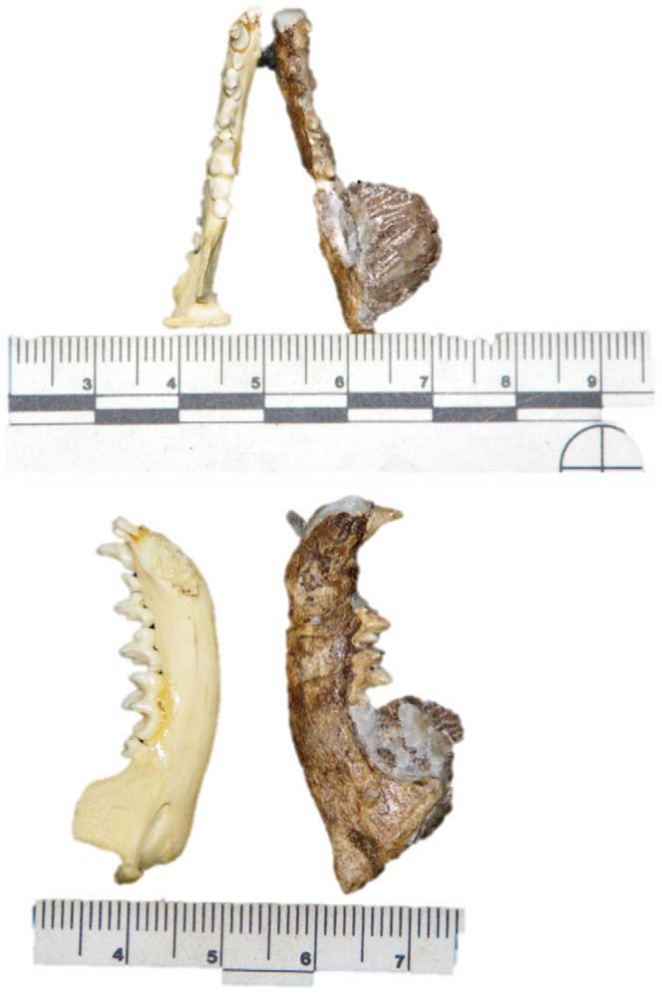
The buccal and occlusal views of UW 88–834, a mandible of cf. *Genetta* compared with the mandible of an extant *Genetta genetta.*

The position and morphology of the P_4_, M_1_, alveoli and roots enable us to tentatively place this specimen in the genus *Genetta.* Overall size limits the assignation to either Herpestidae or Viverridae and as Figure 3 illustrates, the fossil specimen shares overall gross morphologies to extant *Genetta.* In addition, the high middle cusp of the M_1_ as well as the overall narrowness of the M_1_ compared to the M_1_ of herpestids of similar size justifies the assignation to cf. *Genetta* sp. The identification of which species of *Genetta* is represented at Malapa requires considerably more complete material.

Family HERPESTIDAE Bonaparte, 1845

Subfamily HERPESTINAE Bonaparte, 1845

Genus *Atilax* Cuvier, 1826

Atilax mesotes (Ewer, 1956)

Atilax cf. A. mesotes

Material

**Figure 4:**
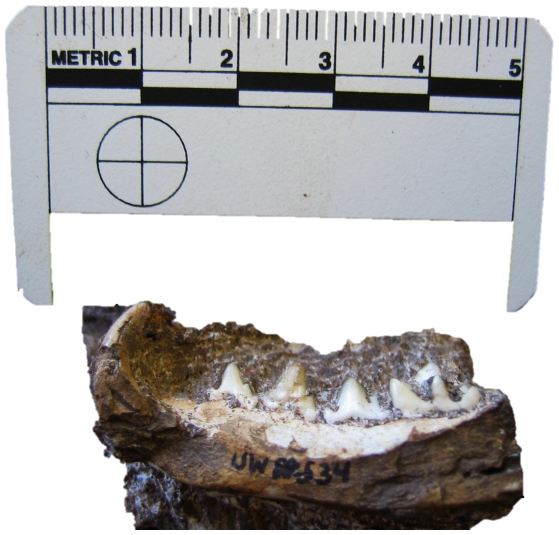
A lateral view of specimen UW 88–534, the mandible of *Atilax* cf. *A. mesotes* recovered from Malapa. This is a morphological match to the Kromdraai specimen KA 86.

UW 88–534, right mandible with left and right canines plus right P_2_-M_1_ (Figure 4).

Comments

This specimen is a morphological match to Ditsong Museum specimen KA-86, previously described as *Herpestes mesotes* [7]. At the time it was indicated that the specimen likely was on the lineage to *Atilax* and in agreement with other authors [8] we have therefore transferred the Kromdraai specimen to this genus.

cf. Herpestidae

Material

UW 88–694, isolated upper canine; UW 88–770, nearly complete sacrum; UW 88–771, distal femur; UW 88–822, partial edentulous skull with left zygomatic arch.

Comments

Comparisons of UW 88–694 to morphological characteristics of both fossil and modern material rule out referral to Viverridae and *Atilax*, but provide a close match in overall size and morphology to specimen TM 32670, an extant yellow mongoose (*Cynictis penicillata*). However, the gross morphology of upper canines show little variability among herpestids, and therefore we have decided to refer the specimen to cf. Herpestidae pending new discoveries.

Based on morphological comparisons with KA 86, specimen UW 88–822 does not belong to the genus *Atilax*. Based on the curvature and size of the cranial vault the specimen closely resembles modern *Rhynchogale melleri,* but does not preserve enough morphological characters to positively refer it to a specific taxon. Therefore we refer it to cf. Herpestidae.

Size comparisons suggest that the mentioned postcranial remains (UW 88–770 and UW 88–771) belong to small herpestids, but lack of enough diagnostic morphological elements forces us to refer them to cf. Herpestidae, though we note a general similarity in size and shape between the fossil sacrum (UW 88–770) and that of *Herpestes ichneumon*.

Family HYAENIDAE Gray, 1821

Subfamily HYAENINAE Gray, 1821

Genus *Parahyaena* Hendey, 1974

Parahyaena brunnea Thunberg, 1820

Material

**Figure 5:**
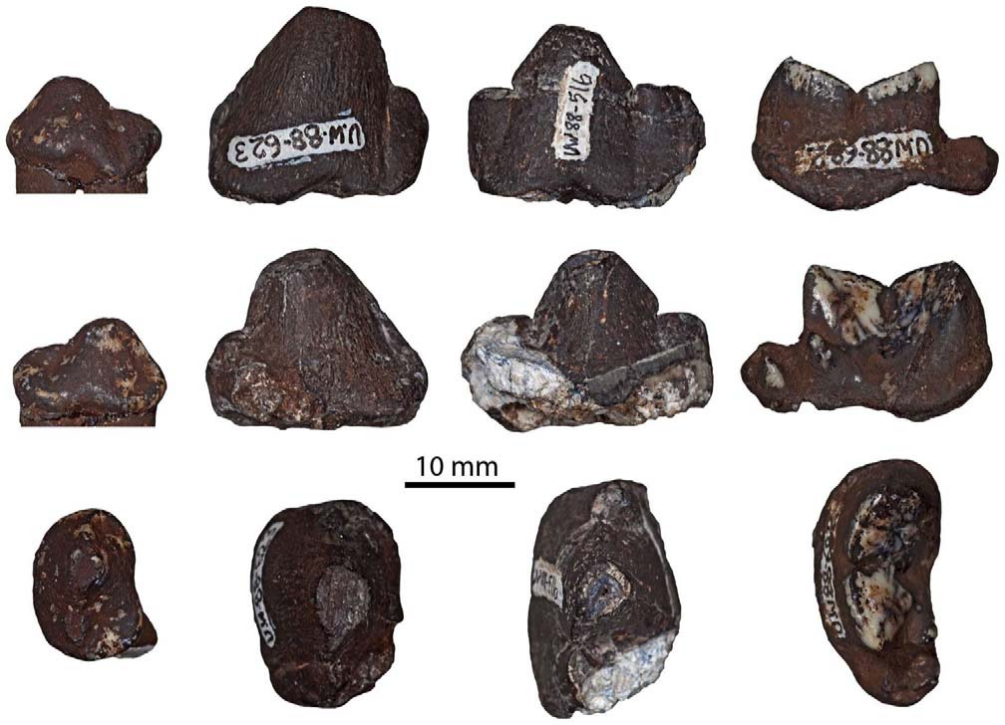
The dentition of fossil *Parahyaena brunnea* recovered from Malapa. From left to right: specimens UW 88–516, UW 88–520, UW 88–623 & UW 88–682 (P_2_-M_1_) in (top to bottom) buccal, lingual and occlusal views.

UW 88–516, crown of left P_4_, L ca. 23.5 W ca. 14.4; UW 88–520, left P_2_, L 14.2 W 10.3; UW 88–521, left P_3_ in two pieces L 19.3 W 12.7; UW 88–623, left P_3_, L 20.2 W 14.2; UW 88–681, right M_1_ trigonid Lt 18.4 W 11.1; UW 88–682 crown of left M_1_, L 23.1 Lt 17.8 W 11.5 (Figure 5).

Comments

**Figure 6:**
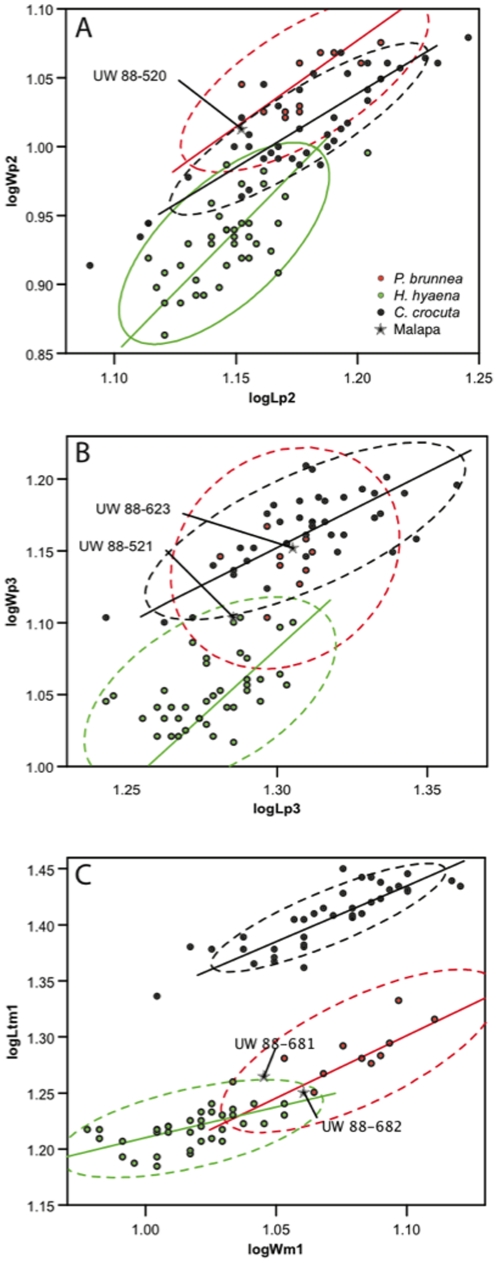
Diagram showing a metric comparison of trigonid length versus width of the lower carnassial (M_1_) of *Parahyaena brunnea* (brown hyaena), *Hyaena hyaena* (striped hyaena), and *Crocuta crocuta* (spotted hyaena), together with two specimens from Malapa, as labeled. A: length and width of P_2_ showing the Malapa specimen UW 88–520 to be similar to *P. brunnea* and *C. crocuta* and different from *H. hyaena* in its greater width. B: length and width of P_3_. One Malapa specimen lies within the ellipses of *P. brunnea* (the sample correlation for this species is not significant and no regression axis can be defined) and *C. crocuta*, while the other lies close to all three ellipses. C: trigonid length of M_1_ versus tooth width. The Malapa specimens lies near the ellipses of *P. brunnea* and *H. hyaena* and far from that of *C. crocuta*.

These specimens are all a good match for extant brown hyaena both in morphology (Figure 5) and metrics (Figure 6). Figure 6A shows that the Malapa P_2_ lies within the confidence ellipses of *P. brunnea* and *C. crocuta* but well outside that of *H. hyaena*, while Fig. 6C shows the M_1_ trigonid to be much shorter relative to tooth width than that of *C. crocuta* but similar in length to *P. brunnea* and *H. hyaena*. The Malapa specimens also markedly differ from extinct hyaenids such as *Pachycrocuta* and *Chasmaporthetes.* The majority of the specimens are likely to belong to a single individual, but the duplication of left P_3_ shows that at least two individuals are involved.

cf. *Parahyaena brunnea*

Material

UW 88–512, MT III, L 89.5 W dist 10.8; UW 88–523, tip of main cusp of left dP^3^; UW 88–524, nearly complete right dP^3^, L 18.1 W 10.2; UW 88–539 left articulated ankle with astragalus, calcaneum, distal tibia, distal fibula; UW 88–577 right distal radius, W dist 33.2; UW 88–778 left proximal humerus shaft; UW 88–782, 88–783 middle and distal phalanges (articulated); UW 88–784, proximal phalanx, L 25.3 W prox 9.4, W dist 8.9; UW 88–787 distal metapodial fragment.

Comments

Parsimoniously, these are specimens that are all highly likely to be *P. brunnea*, as no identifiable craniodental material of any other hyaenid species has been recovered from Malapa. However, we do not have adequate comparative material of deciduous dentitions to make a positive determination of said teeth. In addition postcrania of brown hyaena are also much more difficult to distinguish from those of *C. crocuta* (except by size) than the teeth, especially in the absence of extensive comparative material. Therefore we do not definitively assign these specimens to *P. brunnea*. The presence of deciduous teeth in the sample alongside teeth with some wear suggests the presence of at least three hyaena individuals at Malapa.

Hyaenidae indet.

Material

UW 88–525, nearly complete left lower canine, L 16.5 W 12.1; UW 88–526, fragment of left ventral part of mandibular ramus; UW 88–532 proximal part of middle phalanx; UW 88–537, middle phalanx L 16.9 W prox 10.6 W dist 8.1; UW 88–582, proximal part of right MC V; UW 88–617, nearly complete right upper canine, L ca. 16.5 W ca. 12; UW 88–776, proximal part of caudal vertebra; UW 88–785 proximal phalanx.

Comments

These are all specimens that either due to damage or because of the specific preserved part not providing diagnostic information beyond the family level are left as Hyaenidae indet.

Family FELIDAE Fischer, 1817

Subfamily MACHAIRODONTINAE Gill, 1872

Genus *Dinofelis* Zdansky, 1924

Dinofelis barlowi Broom, 1937

*Dinofelis* cf. *D. barlowi*

Material

**Figure 7:**
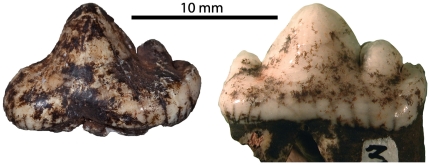
A lateral view of specimen UW 88–627, a P_3_ of *Dinofelis* cf. *D. barlowi* recovered from Malapa compared with P_3_ of *D. piveteaui* from Motsetse (MT-04). Note the difference in the mesial accessory cusps.

UW 88–627, isolated left P_3_. L 13.8 W 7.6 (Figure 7).

Comments

Given the age of Malapa at 1.977 Ma [3], any *Dinofelis* specimen could potentially belong to either *D. barlowi* or *D. piveteaui*. This is the only specimen of *Dinofelis* from Malapa with the potential to discriminate between these two species. The mesial accessory cusp of this specimen is very small or nearly-absent, in agreement with the specimens of *D. barlowi* from Bolt's Farm and Sterkfontein [9]. Specimens identified as *D. piveteaui* from both East and South Africa all have a well developed mesial accessory cusp [9]. The absence of this cusp in UW 88–627 is strongly suggestive of its affinity with *D. barlowi*. At 1.977 Ma, this would make the Malapa specimen the youngest certain record of *D. barlowi*.

cf. Dinofelis sp.

Material

UW 88–513, left femur, distomedial part; UW 88–514, left maxilla fragment with natural molds of canine and carnassial; UW 88–527, right MC II 28.8, W dist 14.9; UW 88–594, left proximal MT IV, W prox 13.3; UW 88–597, left proximal MC II, W prox 16.4; UW 88–598 distal left MC III, W dist 17.9; UW 88–747, articulated right ankle with calcaneum, astragalus, several tarsals, and proximal metatarsal portions; UW 88–773, left distal MC III or IV; UW 88–802, distal right radius; UW 88–803, left distal radius, Wdist 34.0; UW 88–805, iliac blade; UW 88–806, left distal ulna; UW 88–816, right distal MC III or IV; UW 88–820, occipital condyle; UW 88–821, sphenoid.

Comments

The MC I UW 88–527 is of particular interest. It is closely similar to the MC I ER 766K from Koobi Fora (Fig. 19B, 19G [10]) identified as *D. piveteaui* on the basis of association with craniodental remains, in both size and morphology. However, UW 88–527 has a flatter distal end, showing that this specimen does not belong to *D. piveteaui*, and suggesting more power but less mobility of the first digit in the Malapa individual.

The proximal MC II UW 88–597 is similar in size to the homologous element in both *D. aronoki* and *D. piveteaui* from East Africa (Figs. 13C, 19B, 19H [10]) but differs clearly from the latter in the much narrower proximal articular surface. In this UW 88–597 is more like the MC II of *D. aronoki*, again suggesting that the Malapa specimen cannot be attributed to *D. piveteaui*.

The remaining specimens are tentatively identified as *Dinofelis* because they are intermediate in size between *Panthera leo* and *P. pardus*, which is the size interval where the two South African *Dinofelis* species, *D. barlowi* and *D. piveteaui*, are found. In addition, the articulated right ankle UW 88–747 formed part of a pilot study using digital techniques to virtually isolate the individual bones of the specimen without having to prepare out the articulated fossil [11].

Subfamily FELINAE Fisher, 1817

Genus *Panthera* Oken, 1816

*Panthera pardus* Linnaeus, 1758

Material

**Figure 8:**
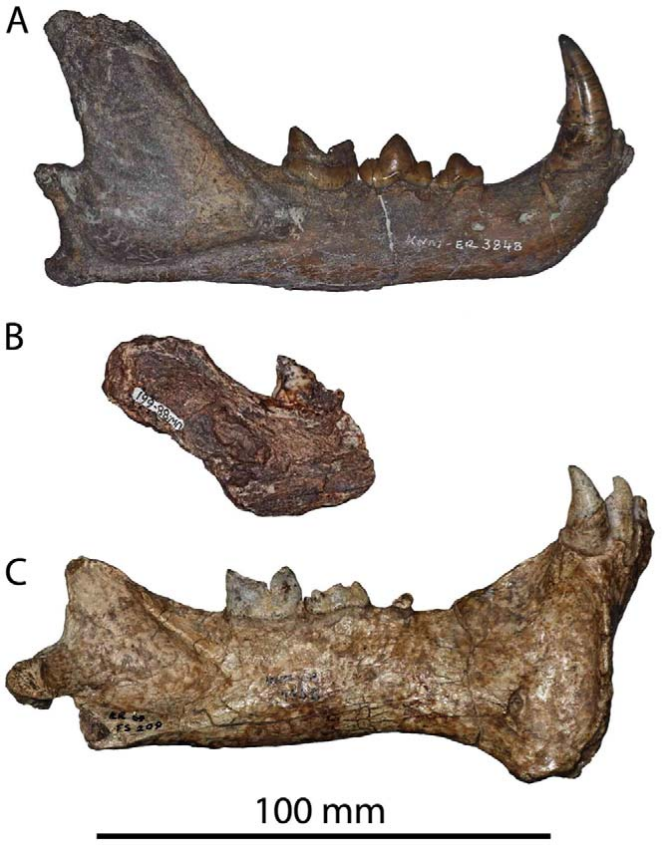
The buccal views of selected felid mandibles. A: KNM-ER 3848 from Koobi Fora, identified unequivocally as *P. pardus* [49] B: Malapa specimen UW 88–661. C: KNM-ER 793B, *M. whitei* [49], [50]. Note the differing shapes of the distal margin of M_1_, as well as the differing angles of the anterior margin of the ascending ramus behind the M_1_.

UW 88–661, right mandibular fragment with broken M_1_. L 16.9, W 8.8. (Figure 8); UW 88–613, left proximal MC IV.

Comments

The mandible is broken anterior to the carnassial roughly in the coronal plane, and the remaining section includes part of the anterior margin of the ascending ramus and masseteric fossa, though the angle, coronoid and condylar processes are absent. The M_1_ is missing the majority of the crown of the paraconid (from the mesial margin of the tooth to the carnassial notch), though the mesial-most point of the tooth is preserved, along with the majority of its cross-section, except for a small fraction (∼0.5 mm) of the mesial outline. The preprotocristid is fairly straight, while the postprotocristid, though mainly vertical, does show a distinct concave curvature. There is a very small remnant of the talonid at the base of the mesial end of the protoconid.

**Figure 9:**
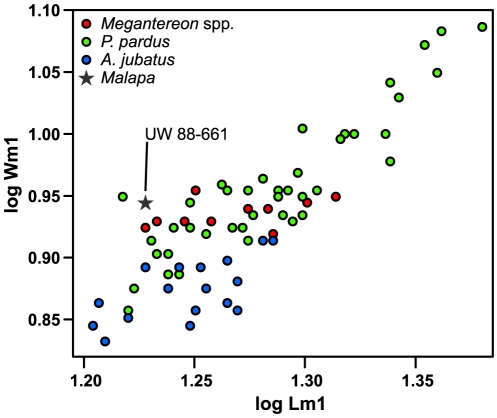
Diagram showing a metric comparison of length versus width of the lower carnassial (M_1_) of *Megantereon* spp. (including both *M. cultridens* and *M. whitei*), *Panthera pardus* (leopard), and *Acinonyx jubatus* (cheetah), together with a Malapa specimen, as labeled. The diagram shows that the Malapa specimen is too wide to belong to *A. jubatus*. *Megantereon* spp. and *P. pardus* overlap almost entirely in size and proportions of M_1_, and therefore the Malapa specimen cannot be assigned to species based on metrics, but only on morphological attributes, as discussed on the text.

Based on its size and the concave distal profile, we ascribe this specimen to *Panthera pardus*. *Dinofelis* spp. and *P. leo* can be excluded based on their large size, and the specimen is also significantly larger than any modern *Felis*, or *Caracal*. This leaves only three possible felid taxa in this size range: *Acinonyx* sp., *Megantereon* sp., and *P. pardus*. *Acinonyx* can be excluded because that taxon has a relatively straight distal margin and a small but highly distinct talonid that is not found in any pantherine. Also, the M_1_ of extant *Acinonyx* is also on average smaller than this specimen, though this may not apply to fossil *Acinonyx* from South Africa. On the other hand, leopard and *Megantereon* spp. lower carnassials are metrically indistinguishable, and the Malapa specimen falls within the range of variation of both (Figure 9). Instead, we consider it unlikely that the specimen belongs to *Megantereon* because of the concave distal profile. Although some older specimens of the genus (e.g., some European *M. cultridens*) show a concave distal margin, none of the African specimens of the genus display anything but a very straight distal border. This can be clearly seen in Fig. 8, which compares the mandibles of fossil *P. pardus* and *M. whitei* to the Malapa fragment. This figure clearly shows how the M_1_ of the Malapa specimen (Fig. 8B) strongly resembles the M_1_ of *P. pardus* (Fig. 8A) and differs from the more ‘upright’ M_1_ of *M. whitei*, with its straighter posterior margin (Fig. 8C). Other early *Panthera* sp., such as the lion-sized *Panthera* from Laetoli show the concave distal M_1_ margin even more clearly [12]. Therefore, we attribute the Malapa specimen UW 88–661 to *P. pardus*.

The MC IV (UW 88–613) is of the size of that of *P. pardus*. In addition, fourth metacarpal of leopards is more transversely slender than that of *Dinofelis*, matching the morphology of this specimen. It is also less oval in lateral view and the proximal articulation is more vertically aligned. Interestingly, it is more similar to the MC IV of *Homotherium*, but the latter is a much larger animal. Thus, in both size and morphology UW 88–613 is closely similar to leopards and we assign the specimen to *P. pardus.*

cf. *Panthera* sp.

Material

UW 88–511, mandible fragment with roots of left and right lower canines and tip of left upper canine; UW 88–595, right unciform; UW 88–638, right unciform.

Comments

The two unciforms are pantherine in morphology, being shorter and less robust than those of the machairodonts *Dinofelis, Megantereon,* and *Homotherium,* as well as less square in lateral view (Fig. 13D–F, 19 L–N [10]). The specimens are too small for *P. leo* but very large for *P. pardus* and we prefer to leave them in open nomenclature.

Genus *Felis* Linnaeus, 1758

*Felis nigripes* Burchell, 1824

Material

**Figure 10:**
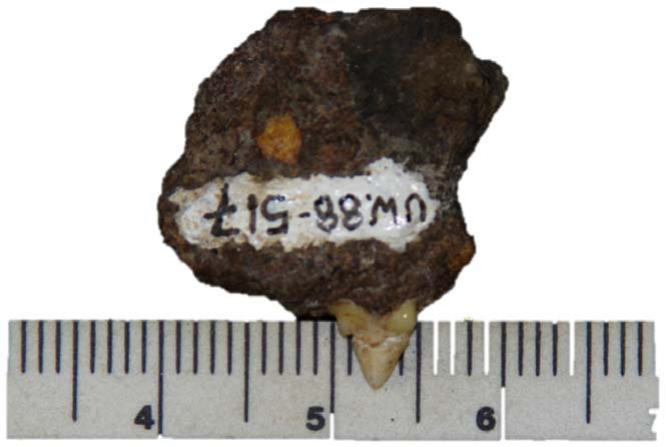
A lateral view of specimen UW 88–517, a P^3^ of *Felis nigripes* recovered from Malapa.

UW 88–517, fragment of left maxilla with P^3^, L 5.1 W 2.4 (Figure 10).

**Figure 11:**
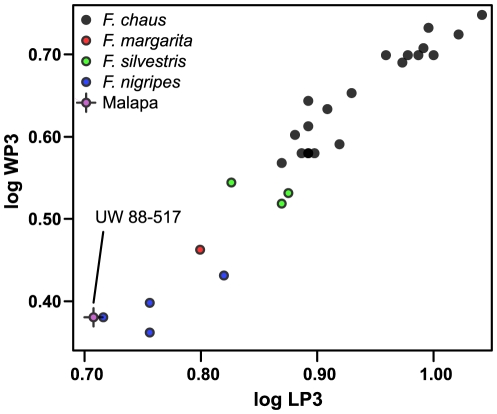
Diagram showing a metric comparison of length versus width of P^3^ of *Felis chaus* (jungle cat), *F. margarita* (sand cat), *F. silvestris lybica* (African wild cat), and *F. nigripes* (black-footed cat), together with a Malapa specimen, as labeled. Despite the small samples of most species, it is very clear that there is a size gradient from *F. chaus* (largest) to *F. nigripes* (smallest), and that the Malapa specimen is at the smaller end of the size range of the smallest species. This, together with morphological attributes discussed in the text, enables us to confidently assign the Malapa specimen to *F. nigripes*.

This very small tooth has a relatively tall main cusp and reduced distal accessory cusp. The mesial accessory cusp and distal cingulum are also small. In metrics the tooth is within the range of variation of a small (N = 5) sample of *Felis nigripes* and outside the range of variation of similar samples of *F. silvestris* and *F. margarita*, the only other African Felidae in this approximate size range (Figure 11). The reduced distal accessory cusp is a diagnostic autapomorphy of *F. nigripes*, as is the relatively tall main cusp [13]. In view of this we refer UW88–517 to *Felis nigripes*.

Felidae indet.

Material

UW 88–533, I_3_, large; UW 88–579, tuber of calcaneum, large size; UW 88–593, caudal vertebra, small; UW 88–614, proximal phalanx, small*-*size; UW 88–616, broken I_3_, large size; UW 88–639, proximal right femur fragment, large size; UW 88–699, pisiform, medium size; UW 88–700, sesamoid, medium size; UW 88–703, proximal phalanx, proximal part, large size; UW 88–704, sesamoid, medium size; UW 88–705, middle phalanx, digit 3, proximal part, large size; UW 88–709, sesamoid, medium size; UW 88–777, long bone shaft fragment, large size.

Carnivora indet.

Material

UW 88–522, root of large I^3^; UW 88–596, large cuboid; UW 88–620, proximal portion of large phalanx; UW 88–696, medium size medial condyle of left femur; UW 88–697, medium size lateral condyle of left femur; UW 88–774, small to medium size sesamoid; UW 88–775, small to medium size sesamoid; UW 88–779, rib fragment of a medium size carnivore; UW 88–786, small to medium size sesamoid; UW 88–789, small to medium size sesamoid; UW 88–804, large magnum; UW 88–968, possible scapula glenoid from a small to medium size carnivore.

Comments

All of the above materials could not, either due to preservation or breakage, be identified beyond a classification as Carnivora. It can be noted that UW 88–697 and UW 88–696 were found in conjunction with one another.

## Discussion

The following discussion will focus on some specimens of particular interest among the Malapa Carnivoran sample. The smaller taxa, such as Herpestidae and Viverridae, will be discussed in detail at a later date as the fossil record of these groups are much less well known than the larger carnivorans, and therefore require extensive primary research before definitive conclusions about taxonomy, systematics, and environmental adaptations can be drawn. Any conclusions drawn here based on these taxa are necessarily preliminary.

### Canidae

Small Canidae are uncommon in the South African Plio-Pleistocene cave sites. The first such material to be described was *Vulpes pulcher* from Kromdraai [14]. Ewer later tentatively referred a specimen from Swartkrans to the same species [15], which is described as somewhat larger than the extant *V. chama* (which is confirmed by the published measurement data). In a later publication [16], Broom described a rather larger canid mandible from Taung as *Vulpes pattisoni*, which is distinguished from *V. chama* by several features in addition to size. Hendey described material of *Otocyon recki* (often referred to the genus *Prototocyon*) from Swartkrans [17], while Turner identified this species as well as *V. chama* from Member 3 of the same site [18]. Most recently, O'Regan and Menter assign some isolated teeth from Drimolen to *V. chama* or Canidae cf. *V. chama* [19]. The Malapa record is thus rare, but in line with the representation of *Vulpes* at other South African cave sites.

The other specimen identified to Canidae is the large distal femur UW 88–838. While the size of the specimen is on par with *L. pictus* the morphological characteristics rule it out as being that species. Further material is needed before we can determine if the specimen belongs to *L. sekowei* or to another, heretofore unknown, form of large Canidae. All of the material attributed to Canidae will be examined in greater detail in a later publication.

### Hyaenidae

The majority of the South African cave sites include material of both *Parahyaena brunnea* and *Crocuta* sp., often in association with extinct forms such as *Pachycrocuta brevirostris* (Kromdraai A, Sterkfontein Mbs. 4 and 5, Makapansgat Mb. 3), *Chasmaporthetes nitidula* (Drimolen, Sterkfontein Mbs. 2, 4, 5, and Jacovec Cave, Swartkrans Mbs. 2 and 3), or *Lycyaenops silberbergi* (Sterkfontein Mbs. 2, 4, and Jacovec Cave) [8]. Although Malapa is an exception at this point, as we can only unequivocally identify material from a single hyaenid species, *P. brunnea*, we believe that this is a sampling issue and predict that with increasing sampling at Malapa, material belonging to at least one more hyaenid species (likely *Crocuta*) will be found. However, it should be noted that hyaenids are commonly the most abundant fossils at these sites, both in terms of the number of specimens and the number of species. The latter is thus far not true at Malapa and it is possible that the difference we see is due to taphonomic processes rather than sample size.

*Parahyaena brunnea* is the only carnivore species identified from Malapa with an MNI greater than one. Since the sample includes both juvenile and adult worn teeth, which include two worn left P_3_, we are confident that we have at least three individuals present. Specimens UW 88–520, 521 and 522 were found in association, as were UW 88–623, 681 and 682. The only other category with an MNI greater than one is cf. *Panthera* sp., which may or may not represent the same species.

### Felidae

The presence of *Felis nigripes* at Malapa represents the first fossil record of this very small (1.3–2.3 kg) cat. The black-footed cat is endemic to the grasslands of southern Africa and generally displays a relatively small home range [20]. The presence of *F. nigripes* thus provides an indication of the habitat around Malapa at the time of deposition.

In the Malapa sample, the only preserved *Dinofelis* tooth is a P_3_, a tooth that presents morphologically diagnostic features. All reported specimens of *D. barlowi* to date have in common the absence of a prominent anterior cusp on the P_3_, whereas all known specimens of *D. piveteaui* from East and South Africa show a strongly developed anterior cusp on this tooth [9], [10] (Figure 7). The absence of an anterior cusp on the *Dinofelis* P_3_ from Malapa strongly suggests its inclusion within the *D. barlowi* hypodigm although definite confirmation of this taxonomic attribution must naturally await the recovery of additional specimens of *Dinofelis* at Malapa. This provisional identification suggests a last appearance datum (LAD) for *D. barlowi* of possibly 1.977 Ma, younger than previously suggested. This younger age has implications for the evolution of the genus *Dinofelis* in Africa, more specifically bearing on the origin of *D. piveteaui*, the last known species of the genus. *Dinofelis piveteaui* is known from both East and South Africa [10], [21], [22] but the details of its evolution are contentious [9], [10], [19]. Two rival scenarios are available. *Dinofelis piveteaui* may have evolved in South Africa from the earlier South African species *D. barlowi*, and subsequently migrated to East Africa [19], [22]. Alternatively, *D. piveteaui* evolved in East Africa from the older *D. aronoki* and subsequently migrated to South Africa [10]. Choosing between these hypotheses involves making progress along several lines of inquiry, including better dated localities, particularly in South Africa, and better and more complete character analyses of the existing material.

At present, in the African continent, at least six *Dinofelis* species may be recognized, including, from chronologically oldest to most recent: *D.* cf. *D. diastemata*, *D. petteri*, *D. aronoki*, *D. darti*, *D. barlowi* and *D. piveteaui*, although there is no general consensus in this classification [9], [10], [15] The genus *Dinofelis* has sometimes been placed in the subfamily Felinae along with modern cats and sometimes in the subfamily Machairodontinae, sabertooth cats. The main source of this confusion lies in its dental morphology. The upper canines are not highly mediolaterally compressed as they are in typical machairodonts such as *Smilodon* and *Homotherium*, yet they lack the longitudinal (‘feline’) grooves characteristic of most extant cat species, especially the larger members of the genus *Panthera*, with which *Dinofelis* is most often compared. A recent study resolves this question by showing that *Dinofelis* should be placed in Machairodontinae on the basis of derived characters such as the size differential between upper and lower canines, reduction of anterior cheek dentition, and a large pit superomedial to the trochlear notch of the ulna [10]. By this interpretation, for a large part of the genus' existence species *Dinofelis* played an ecological role similar to that played by species of the genus *Panthera* today.

Given this ecological context, the evolution of *D. piveteaui* is of particular interest, as it represents an evolution away from the “*Panthera*-like” paradigm followed by its ancestors, towards a distinctly more machairodont morphology, with more compressed upper canines, further reduced anterior cheek teeth, and a more machairodont skull and postcranium. This change in an evolutionary trend almost certainly represents a response to other changes in the carnivore guild, which makes understanding the evolution of *D. piveteaui* one of the keys to understanding the turnover in the carnivore guild in Africa between 2.0 and 1.5 million years [24].

*Dinofelis barlowi* is found at Sterkfontein, being restricted to Members (Mb) 2 (ca. 2.8–2.6 Ma) and Mb. 4, [25], [26], [27], [28]. A previous report of *D. barlowi* at Sterkfontein Mb. 5 [25] is an annotation error (A. Turner, personal communication to RL, March 29, 2011). This taxon has also been reported from a series of pits at the site of Bolt's Farm [23], [29], and although it is generally believed that parts of this site may exceed 2.0 Ma in age, dating remains problematic. Unconfirmed reports have indicated that *D. barlowi* was also recovered from Gladysvale [30], but this claim remains contentious and in any case, no stratigraphic information is available for this possible identification. *D. barlowi* remains as a South African endemic species. Its East African counterpart is *D. aronoki*, which is known from a number of sites in the Turkana Basin dating between ca. 3.0 Ma and <1.6 Ma [10].

In East Africa, *D. piveteaui* has been reported from Koobi Fora, and Kanam East in Kenya [23], [31], and Konso Gardula in Ethiopia [32]. These sites are temporally constrained to between 1.64 and 1.0 Ma, although the younger date assigned needs further confirmation. In South Africa, *D. piveteaui* is presently recognized from Kromdraai (which includes the type specimen, KA 61), Motsetse [9], [33], Gladysvale [9], and more recently from the site of Drimolen [19]. Other *Dinofelis* specimens recovered from the caves of Swartkrans, Coopers, and the Kromdraai B deposits [9], [10], [18] have not been conclusively assigned to species. With the exception of Motsetse, whose age was largely inferred based on the *D. piveteaui* remains, the ascribed faunal-based ages of Kromdraai A and Drimolen are ca. 2.0–1.5 Ma [34], [35], [36] The Gladysvale *D. piveteaui* specimen derives from material without precise stratigraphic context [30].

The picture that emerges from these taxonomic identifications is that South African fossil localities older or around 2.0 Ma record the presence of *D. barlowi*, while the chronologically younger sites are associated with *D. piveteaui* remains. The earliest record of *D. piveteaui* in East Africa, on the other hand, dates to less than1.64 Ma, which is the lower boundary datum of the Okote Mb. of the Koobi Fora Fm. [37]. Thus, either *D. piveteaui* evolved earlier in East Africa than its present first appearance datum (FAD) implies (i.e., before 1.64 Ma), or the ages of Kromdraai and Drimolen may be best constrained at the younger end of their age range as *D. piveteaui* has been recovered from both sites. However, if *D. piveteaui* evolved in South Africa from its putative ancestor *D. barlowi* [23], then it may not be unreasonable to expect the recovery of intermediate forms between 2.0 and 1.5 Ma [19]. *D. piveteaui* specimens from Kromdraai include a well preserved skull with teeth (KA 61), a P_3_ (KA 62) and an M_1_ (KA 63), which together with lower dentition of the Motsetse specimens provide important information in assessing differences between *D. barlowi* and *D. piveteaui* [9], [10]. Most notably, the expanded P^4^ metastyle and increase in length of M_1_ contrast with the morphology of *D. barlowi* characterized by relatively smaller P^4^ metastyles and shorter M_1_ [9].

The *D. barlowi* specimen from Malapa at 1.977 Ma therefore bears on the evolution of *Dinofelis*, increasing the plausibility of a South African origin by lessening constraints on the FAD of *D. piveteaui*. Thus, with a *D. barlowi* LAD of over 2 Ma, the FAD of *D. piveteaui* must be close to the lower age limit of the sites at which it has been found, while a younger LAD for *D. barlowi* such as that represented by Malapa allows for a *D. piveteaui* FAD well within the suggested age range for sites such as Drimolen. On the other hand, *D. piveteaui* shares with *D. aronoki* morphological traits that it does not share with *D. barlowi*. Thus, even though Malapa represents a step forward, only additional well-preserved fossils from well dated geological contexts, especially in South Africa, can resolve the issue.

The Carnivora identified from Malapa to date suggest the presence of two or more microhabitats in proximity to the cave. Today, *F. nigripes* is endemic to southern Africa and associated with the more arid regions that provide some cover in the form of grasses and scrub [38], [39]. With home ranges recorded from 9.99 km^2^ (females) to 20.69 km^2^ (males) [40], the presence of *F. nigripes* at Malapa indicates that at least some of the region surrounding Malapa consisted of grasses, brush and scrub. The identification of *Vulpes* cf. *V. chama* supports the hypothesis of open grasslands as these, along with scrub and desert, are the preferred habitats of *V. chama* today [39]. We also note that, with few exceptions the family Canidae and the genus *Felis* today are both associated with open habitats [39]. *Parahyaena brunnea* historically has been associated with dry open habitats [39], [41], [42], and has not been documented to inhabit closed, forest-like environments. *Panthera pardus* today is exceptionally eurytopic and tolerant of a variety of habitats [39] and thus the presence of *P. pardus* is of little use with regard to interpreting the palaeoenvironment associated with Malapa. The presence of the genus *Genetta* is problematic as *G. genetta* today inhabits open regions whereas *G. tigrina* today is found in more closed environments [39]. The other two taxa, *Atilax* and *Dinofelis*, are clear indicators of more closed habitats. In the case of *Atilax*, the modern species has a strong affinity for wet to very wet habitats along streams or lakes, though we don't know if that applies to the fossil species as well. The brachial index of *Dinofelis* spp. indicates closed habitat adaptation [9] in line with paleoecological interpretations from other *Dinofelis* sites. In addition, yellowwood (*Podocarpus* sp.) along with other forest plant species have also been identified from pollen recovered from a coprolite at Malapa [43]. Thus, plant species indicate a moist, forest environment that corroborates the information from two of the identified carnivore species while open grasslands are indicated by two other identified carnivore species.

It is also worth noting that, if the geological interpretations are correct in that the Malapa assemblage is attributed to a death trap caught at a single moment in time, the social behaviours of the carnivores identified to date may be reflected in the assemblage. Even though the assemblage is small, and it is still early in excavations, the MNI's reflect the social behaviours in that *P. pardus, F. nigripes, V. chama, Genetta* and *Atilax* all have an MNI of one and today are all solitary in nature while *Parahyaena brunnea* (MNI of 3) is a clan oriented social species. The modern analogues of all of the carnivores identified to date are also territorial, thus unlike bovids which can roam far in search of browse or grazing, the carnivores would remain within the territories (unless the specimens we recovered happen to be younger animals looking to establish a territory) suggesting that both the open grasslands and the forested woodlands existed within ca. 20 km^2^, the smallest of the territories (*F. nigripes*) identified to date.

We have described the carnivore assemblage associated with the *Au. sediba* remains from Malapa, South Africa [3], [4], [5], [44], [45], [46], [47]. The analysis of this assemblage and its chronological age provide clues to the evolution of several mammalian taxa, with possible LAD for *D. barlowi* and the FAD for *F. nigripes*. The Malapa assemblage currently lacks extinct genera such as Megantereon (contrary to initial reports [4]), Chasmaporthetes or Pachycrocuta. Based on the carnivore remains described here, we suggest that the environments of *Au. sediba* are characterized by presence of relatively closed habitats with nearby open grasslands. This interpretation is in keeping with that described based on plant species [43] and bovid taxa [4] represented at the site.

## Methods

All specimens were identified using reference collections housed at the Institute for Human Evolution (IHE) and Bernard Price Institute (BPI), University of the Witwatersrand as well as material housed at the Ditsong Museum of Natural History in Pretoria (formerly known as the Transvaal Museum), with other measurement and photographic data provided by the personal files of LW. All measurements were taken with digital callipers to the nearest 1/10 millimetre (mm) and follow von den Driesch [48] unless otherwise noted. Tooth lengths (L) are mesial-distal and widths (W) are buccal-lingual. Both BK and LW took measurements. Other abbreviations: MNI: minimum number of individuals; NISP: number of individual specimens.
